# Feeding sunflower cake from biodiesel production to Santa Ines lambs: Physicochemical composition, fatty acid profile and sensory attributes of meat

**DOI:** 10.1371/journal.pone.0188648

**Published:** 2018-01-05

**Authors:** Anny Graycy Vasconcelos de Oliveira Lima, Ronaldo Lopes Oliveira, Thadeu Mariniello Silva, Analívia Martins Barbosa, Thiago Vinicius Costa Nascimento, Vinicius da Silva Oliveira, Rebeca Dantas Xavier Ribeiro, Elzânia Sales Pereira, Leilson Rocha Bezerra

**Affiliations:** 1 Animal Science Department, Federal University of Bahia, Salvador, Bahia, Brazil; 2 Department of Preventive Veterinary Medicine and Animal Production, Federal University of Bahia, Salvador, Bahia, Brazil; 3 Department of Animal Science, Federal University of Ceara, Fortaleza, Ceara, Brazil; 4 Department of Animal Science, Federal University of Piaui, Bom Jesus, Piaui, Brazil; Leibniz-Institut fur Pflanzengenetik und Kulturpflanzenforschung Gatersleben, GERMANY

## Abstract

The aim of this study was to determine the effect of the inclusion of sunflower cake in the diets of lambs on meat quality. Forty male, uncastrated Santa Ines lambs with an initial average body weight of 20.9 ± 0.41 kg were used in a completely randomized design with four treatments. The lambs were fed 500 g/kg hay and 500 g/kg concentrate (corn, soybean meal, urea, ammonium sulfate and sunflower cake) in their diet, and the experimental treatments were 0, 10, 20, and 30% sunflower cake inclusion (DM basis). The inclusion of sunflower cake in the diet did not affect (*P* > 0.05) the content of ash and protein, overall acceptance, or sensory aroma of the lamb meat. Total lipids and moisture content of the *longissimus lumborum* muscle increased linearly (*P* ≤ 0.05) with sunflower cake inclusion. The C16:0, ΣSFA, ΣMUFA:ΣSFA ratio, Δ–9 desaturase enzyme and atherogenicity index in the *longissimus lumborum* muscle decreased linearly (*P* ≤ 0.05) with sunflower cake inclusion in lamb diets, whereas a linear increase occurred (*P* ≤ 0.05) in C12:0, C18:0, ΣMUFA, ΣPUFA, ΣPUFA:ΣSFA and ΣPUFA:ΣMUFA ratios, Σn–6, Σn–3, desirable fatty acids, h:H index, elongase activity, and flavor, tenderness and juiciness sensory qualities in lamb meat. Among the panelists, the highest preference (35.9%) was for meat with 30% sunflower cake inclusion in the diet. Sunflower cake up to 30% of total DM can be included in the diet of Santa Ines lambs, because lipid nutritional quality and the sensory qualities of the lamb meat increase, in addition to improvement in nutraceutical compounds, such as the CLA content, and in the AI, PUFA:SFA and PUFA:MUFA ratios, desirable fatty acids content and h:H ratio, which are beneficial to the health of consumers.

## Introduction

Cakes produced from biodiesel can be used in the diets of ruminants, because these cakes increase the energetic density of the diet, are sources rich in polyunsaturated fatty acids and when added at moderate levels, improve the quality of the meat that is produced [1–3]. In particular, sunflower (*Helianthus annuus*) seeds are outstanding, because the seed oil is noble with great nutritional value, rich in polyunsaturated fatty acids (PUFAs) and primarily linoleic acid (68 g/100 g of meat fat) in lipid composition [4,5].

Linoleic acid (C18:2 n*–*6) is an essential fatty acid that is responsible for important functions in human health, although this fatty acid increases oxidation and n*–*6 concentration [6,7]. Diets rich in C18:2 n*–*6 provide a greater supply of PUFAs that are mostly subjected to the mechanism of biohydrogenation that occurs within the rumen. This mechanism is the primary determinant of the fatty acid profile in meat. Intermediate products of the biohydrogenation process, such as rumenic acid (C18:2 *cis–*9, *trans–*11) and vaccenic acid (C18:1 *trans*-11), are the primary fatty acids (FAs) that improve the lipid profile of the final product [8,9].

Endogenously, vaccenic acid can be converted to conjugated linoleic acid (CLA) by the Δ–9 desaturase enzyme. This fatty acid is deposited in adipose tissue, which provides a better fatty acid profile that is beneficial and improves the meat quality [10,11]. The benefits occur primarily because meat from ruminants is considered rich in saturated fatty acids (SFAs) that are responsible for increasing plasma cholesterol, cardiovascular diseases and atherosclerosis [7].

Thus, the primary way to change the fatty acid composition of ruminant meat is the use of lipid sources that cause non-negative or minimal effects in the rumen environment and simultaneously supply precursors for CLA synthesis in the rumen and/or in body tissues. Therefore, to provide consumers with better-quality meat, we hypothesized that sunflower cake, which is a source of linoleic acid, would increase the conjugated linoleic acid content and other nutraceutical compounds and decrease the SFA content, thereby improving meat quality. In this study, the effects of sunflower cake inclusion in lamb diet on meat quality were evaluated through analyses of the physicochemical composition, fatty acid profile and sensory attributes of the *longissimus lumborum* muscle of Santa Ines lambs.

## Materials and methods

### Ethical considerations and study location

This study was conducted at the Federal University of Bahia in strict accordance with the recommendations of the Guide for the Care and Use of Agricultural Animals in Research and Teaching and was approved by the Committee on the Ethics of Animal Experiments of the Federal University of Bahia, Bahia State, Brazil (Protocol Number 02/2014). This research was submitted and approved also by the Brazil Platform for the Ethical and Methodological aspects according to the guidelines established in Resolution 466/2012 and complementary to the National Health Council, approved by the Research Ethics Committee of the Federal University of Piauí - UFPI. Before participating in the assessment of meat sensorial attributes, the participants signed an informed consent form. The responsible researcher signed the terms of responsibility ensuring that the panelist identification was preserved.

#### Animals, treatments, diets and general procedures

Forty male, uncastrated Santa Ines lambs that had an initial average body weight (BW) of 20.9 ± 0.41 kg were used in a completely randomized design with four treatments and ten repetitions. The experimental treatments consisted of 0, 10, 20, and 30% sunflower cake inclusion in diets (DM basis). The total mixed rations (TMRs) contained Tifton-85 hay with a forage particle size of approximately 5 cm and forage to concentrate ratio of 50:50. The lambs were fed twice per day (0900 and 1600h) with a total mixed ration, and the diet was formulated to supply the nutritional requirements of late maturity lambs with a gain of 250 g/day as recommended by the National Research Council (NRC) [12].

The animals were housed in a covered shed in 1.0-m^2^ individual pens with *ad libitum* access to water and the experimental diets. Lambs underwent an adaptive period of 15 days. In the adaptation period, all animals were treated against internal and external parasites with Ivermectin (Ivomec gold; Merial, Salvador, Bahia, Brazil) and vaccinated against clostridiosis using Polivalente (Sintoxan; Merial). The experimental period was 71 days. The ingredient proportions with the chemical composition of experimental diets are presented in Table 1.

**Table 1:** Ingredient proportions and chemical composition of experimental diets.

Item	Sunflower cake (% DM)			
	0	10	20	30
Ingredient (% DM)				
Ground corn	26.0	20.7	15.3	10.0
Soybean meal	22.0	17.3	12.7	8.00
Sunflower cake	0.0	10.0	20.0	30.0
Mineral mixture*	1.50	1.50	1.50	1.50
Urea + ammonium sulfate^†^	0.50	0.50	0.50	0.50
Tifton-85 hay	50.0	50.0	50.0	50.0
Chemical composition (% DM)				
Dry matter (% as fed)	87.5	87.5	87.5	87.4
Ash	6.25	6.48	6.71	6.94
Crude protein	18.8	18.5	18.1	17.8
Ether extract	2.41	3.67	4.94	6.21
Neutral detergent fiber ap^¥^	41.2	43.3	45.4	47.5
Acid detergent fiber	22.0	23.9	25.7	27.6
Neutral detergent insoluble nitrogen (% CP)	33.8	34.1	34.5	34.9
Acid detergent insoluble nitrogen (% CP)	1.80	2.05	2.30	2.55
Lignin	3.08	3.75	4.42	5.09
Cellulose	18.9	20.1	21.3	22.5
Hemicellulose	19.2	19.4	19.7	19.9
Non-fibrous carbohydrate	32.2	29.0	25.7	22.5

*Guaranteed levels (for active elements): 120 g calcium, 87 g phosphorus, 147 g sodium, 18 g sulfur, 590 mg copper, 40 mg cobalt, 20 mg chromium, 1,800 mg iron, 80 mg iodine, 1,300 mg manganese, 15 mg selenium, 3,800 mg zinc, 300 mg molybdenum, and maximum 870 mg fluoride. Solubility of phosphorus citric acid, 2 to 95%.

^†^Mixture of urea and ammonium sulfate at a ratio of 9:1.

^¥^Corrected for ash and protein.

### Chemical analyses

Samples of ingredients, refusals, and feces were pre-dried in a forced-ventilation oven at 55°C for 72 h and ground in a Wiley knife mill. Ingredients and refusals were ground with a sieve size of 1 mm, and a sieve size of 3 mm was used for feces. Samples were analyzed according to the methods of AOAC [13] for dry matter (DM; method 967.03), ash (method 942.05), crude protein (CP; method 981.10), and ether extract (EE; method 920.29) contents.

Neutral detergent fiber (NDF) and acid detergent fiber (ADF) were determined using the methods of Van Soest et al. [14] with the modification proposed by Senger et al. [15] to use an autoclave. The autoclave temperature was set to 110°C for 40 min. The NDF residue was incinerated in an oven at 600°C for 4 h, and the protein correction was determined by subtracting the neutral detergent insoluble protein. Neutral detergent insoluble nitrogen (NDIN) and acid detergent insoluble nitrogen (ADIN) contents were determined according to the methodology of Licitra et al. [16]. Acid detergent lignin (ADL) was determined according to AOAC [13], and ADF residue was treated with 72% sulfuric acid. The non-fiber carbohydrates (NFC) were calculated according to Hall [17]: NFC = 100 –[(CP–CP from urea + urea) + NDF + EE + Ash, using the value of NDF that was corrected for ash and protein.

### Slaughtering procedure and obtaining the *longissimus lumborum* muscle

After 18 h of fasting, the animals were weighed and slaughtered. In the slaughtering procedure, the animals were stunned with proper equipment (Dal Pino, Santo André, SP, Brazil) that promoted electronarcosis (220 V, 1.5 A for 10 s). Then, the carcasses were suspended and bled from the jugular vein and carotid artery before they were skinned and eviscerated following the recommendations of the Federal Inspection Service (S.I.F.) that are advocated by the Ministry of Agriculture Livestock and Food Supply [18].

The head and feet were removed, and the carcasses were placed in a cold chamber (4°C) for 24 h. The carcasses were cut, and the *longissimus lumborum* muscle was used to evaluate meat quality based on physicochemical composition, fatty acid profile and sensory characteristics.

### Physicochemical composition of the *longissimus lumborum* muscle

The pH was measured 24 h after slaughter in the *longissimus lumborum* muscle using a Mettler M1120x pH meter (Mettler Toledo International Inc., Columbus, Ohio, United States) according to AOAC [19] procedures. Then, an average was calculated as the pH value of the muscle.

Cooking weight loss (CWL) of the *longissimus lumborum* muscle was measured in two samples that were 2.5 cm thick. The weight of the samples was recorded before and after cooking. The samples were trimmed of subcutaneous fat and cooked on an electric grill. A stainless-steel thermocouple (Gulterm 700; Gulton do Brazil) was placed into the geometric center of each sample to check and record the internal temperature. The samples were cooked until the internal temperature reached 71°C; the samples were then removed from the grill (George Foreman Jumbo Grill GBZ6BW, Rio de Janeiro, Rio de Janeiro, Brazil), placed into a plastic bag and cooled to 10°C in an ice water bath. The cooking weight loss of each sample was obtained, expressed in g/kg, as the difference between the weights before and after cooking, and these samples were then equilibrated at 4°C overnight for instrumental texture analysis conducted according to the method of the American Meat Science Association [20].

On the following day, the samples were brought to room temperature before Warner–Bratzler Shear Force (WBSF) analysis. At least three cores 1.27 cm in diameter and 2.0 cm in length that were parallel to the muscle fibers were removed from each sample using a cork borer. Each core was sheared perpendicular to the fiber direction. The WBSF was measured by a texture analyzer (Texture Analyzer TX-TX2; Mecmesin, Nevada, United States) fitted with a Warner–Bratzler-type shear blade with load of 25 kgf (kilogram-force) and a cutting speed of 20 cm/min [21].

The evaluation of meat color was conducted on the back section using a transverse cut, and the meat was exposed to the atmosphere for 30 min before reading the oxygenated myoglobin level. Then, as described by Miltenburg et al. [22], after 30 min, the color values were measured at three different points on the inner surface of the muscle, and the average of triplicate measures was subsequently calculated separately for each animal: L*, the index related to luminosity (L* = 0, black; = 100, white); a*, the index that ranges from green (−) to red (+); and b*, the index that ranges from blue (−) to yellow (+). These measurements were performed using a MINOLTA CR-10 (Konica® Minolta, Osaka, Japan) calibrated blank tile colorimeter with the CIELAB system, which considers the L*, a* and b* coordinates responsible for brightness (black/white), red content (green/red) and yellow content (blue/yellow), respectively. The saturation index (Chroma;) was determined using a* as (a) and b* as (b) data according to the formula: $Chroma=\sqrt{\left(a^{2}\right)+\left(b^{2}\right)}$. Additionally, Hue angle (H°) as hab = arctangent (b/a) was determined according to Hunt and King [23].

Determination of moisture, mineral and crude protein content followed AOAC [19] recommendations. To determine the fatty acid profiles of the meat and diet samples, lipid extract was obtained using a technique described by Bligh and Dyer [24] with adaptations and using 2:1 chloroform and methanol as solvents. Cholesterol content was obtained according to the methods of Saldanha et al. [25] using a LABTEST® Diagnostic SA enzymatic kit.

The lipids extracted from samples of diets and *longissimus lumborum* muscles were derivatized according to the method described by Hartman and Lago [26].

### Fatty acid profile of the *longissimus lumborum* muscle

To determine the fatty acid profile, the lipids previously extracted from the *longissimus lumborum* muscle and diets (Table 2) were converted to fatty acid methyl esters (FAME). The FAME were prepared using a solution of methanol, ammonium chloride and sulfuric acid, following the procedure described by Hartman and Lago [26].

**Table 2:** Dietary fatty acid (g/100 g FAME) composition of experimental sunflower cake.

Fatty acids (g/100 g FAME)	Sunflower cake (% DM)			
	0	10	20	30
ΣSaturated fatty acids (SFA)	29.6	19.6	7.96	16.2
C10:0	0.03	0.03	0.01	0.01
C12:0	0.03	0.01	0.01	0.03
C14:0	0.07	0.08	0.07	0.08
C15:0	0.05	0.05	0.04	0.03
C16:0	24.7	14.4	2.70	11.2
C17:0	0.12	0.09	0.20	0.06
C18:0	4.23	4.72	4.51	4.37
C20:0	0.33	0.06	0.07	0.11
C24:0	0.12	0.19	0.35	0.33
ΣMonounsaturated fatty acids (MUFA)	44.6	47.9	54.0	49.2
C10:1	0.01	0.01	0.01	0.01
C17:1	0.06	0.13	0.30	0.53
C16:1 *cis*–9	0.14	0.13	0.12	0.12
C18:1 *trans* 8–9	0.11	0.04	0.03	0.03
C18:1 *cis*–9	43.0	46.8	52.4	47.6
C18:1 *cis*–11	1.21	0.55	0.89	0.64
C20:1	0.04	0.13	0.18	0.16
C24:1	0.10	0.10	0.10	0.10
ΣPolyunsaturated fatty acids (PUFA)	25.8	32.5	38.1	34.6
C18:2 *cis* 9–12	24.6	31.5	36.8	32.8
C18:2 *trans* 10–12	0.46	0.55	0.54	0.78
C18:3 n–3	0.30	0.32	0.41	0.54
C20:3 n–6	0.01	0.01	0.03	0.021
C22:2	0.12	0.01	0.01	0.01
C20:5 n–3	0.18	0.01	0.01	0.01
C22:6 n–3	0.15	0.19	0.29	0.37

To identify the lipid profiles, samples were analyzed using a gas-mass spectrometer (GCMS-QP2010 SE; Tokyo, Japan) equipped with a RT-x Wax Polyethylene Glycol column (30 m long × 0.25 mm internal diameter × 0.25 μm film thickness). The column oven temperature was as follows: initial temperature of 100°C, increased at 5°C/min to 190°C and at 5°C/min to 220°C and then maintained for 5 min at a rate of 2°C/min. Finally, the temperature was raised to 240°C at a rate of 5°C/min maintained for 5 min.

Helium (He) was used as the carrier gas at a flow rate of 1 mL/min, and the split ratio was 1:30. The temperature of the injector and detector used was 250°C. The quantification of the methyl esters of fatty acids was based on the normalization of the area [27]. The samples and the standard were injected into the chromatograph together with an internal standard (Methyl Palmitate; SIGMA-ALDRICH, St. Louis, Missouri, United States), and the internal standard concentration used was 25.5 mg/L. The FAME were identified by a comparison of the FAME retention times with those of authentic standards (FAME Mix, C4-C24; SIGMA-ALDRICH, St. Louis, Missouri, USA). To quantify the fatty acid methyl esters, a response factor was generated for each fatty acid based on the standard sample. Rigor was ensured in all procedures for quantification of fatty acids, including attention to quality of standards, weighing, pipetting, use of calibrated and clean material, and evaporation of solution solvents and concentration of solutes, among others. The experimental response factors were close to the theoretical factors. The results were quantified by normalizing the areas of the methyl esters and are expressed as g/100 g fatty acid methyl esters (FAME).

The totals for saturated fatty acids (SFA), monounsaturated fatty acids (MUFA) and polyunsaturated fatty acids (PUFA), in addition to the ∑MUFA:∑SFA, ∑PUFA:∑SFA, ∑PUFA:∑MUFA, and ∑n6:∑n3 ratios, were calculated from the identified fatty acid profiles. To evaluate the nutritional quality of the lipid fraction of the *longissimus lumborum* muscle, the Atherogenicity Index (AI) was calculated as PUFA:MUFA $AI=\left[\left(C_{12:0}+\left(4\times C_{14:0}\right)+C_{16:0}\right)\right]/\left(\sum MUFA+\sum n6+\sum n3\right)$ according to the method of Ulbricht and Southgate [28]; the hypocholesterolemic and hypercholesterolemic (h:H) fatty acids ratio was calculated as $h:H=\left(C_{18:1c\;is\;9}+C_{18:2\;n6}\right)/\left(C_{14:0}+C_{16:0}\right)$ according to Arruda et al. [29]; and the desirable fatty acids (DFA) were calculated as $DFA=\left(\sum \;MUFA+\sum \;PUFA+C_{18:0}\right)$ according to Rhee [30].

The activities of Δ9-desaturase C16 (D9C16), da Δ9-desaturase C18 (D9C18) and elongase were estimated according to Smet et al. [31] with the following equations: $\Delta_{9}C_{16}=\left[C_{16:1}\;/\;\left(C_{16:0}+C_{16:1}\right)\right]\times 100$; $\Delta_{9}C_{18}=\left[\left(C_{18:1cis9}\right)/\left(C_{18:0}+C_{18:1cis9}\right)\right]\times 100$; and $Elongase=\left[\left(C_{18:0}+C_{18:1cis9}\right)\;/\;\left(C_{16:0}+C_{16:1}+C_{18:0}+C_{18:1cis9}\right)\right]\times 100$.

### Sensory attributes

The sensory characteristics of the *longissimus lumborum* were evaluated using a panel of 75 consumers [20]. All panelists were members of the animal science department and included 40 women and 35 men in an age group between 19 and 55 years old accustomed to eating lamb meat. The *longissimus lumborum* muscle samples (30 g) were grouped by treatment (n = 4), placed on an electric grill (George Foreman Grill Jumbo GBZ6BW, Rio de Janeiro, Brazil) and cooked until the geometric center of the samples (duplicate) reached 71°C. The cubes were transferred to a water bath (75°C) covered with aluminum foil to keep them heated and prevent the loss of volatile aroma compounds until the sensory analyses were conducted. No salt or condiments were added.

Tests were performed between 0900 and 1200h, and the panelists were in individual cabins in the sensory panel room. Each panelist received samples (duplicates) of the four treatments totaling eight coded 3-digit cubes without any mention of the levels of sunflower cake used. Water and cream cracker-type biscuits to remove the aftertaste between tastings accompanied the meat samples. The sensory attributes (overall acceptance, aroma, flavor, tenderness and juiciness) were recorded using a hedonic scale of nine points (The scores ranged from 1 to 9 as follow: 1 dislike extremely to 9 like extremely) according to the AMSA [20]. A preference-ordering test was used, and for this test, a token was used for the panelists to order the samples from the least preferred to the most preferred.

### Statistical analyses

The experimental design was completely randomized with 4 treatments and 10 replications. The statistical model used was as follows:

$$Y_{ij}=\mu +si+e_{ij}$$

where Y_ij_ = observed value; μ = overall mean; si = effect of sunflower cake concentration in diet; and e_ij_ = effect of experimental error.

Polynomial contrasts were used to determine the linear and quadratic effects of the different levels of treatment. The command PROC GLM of the SAS 9.1 [32] statistical software package (SAS Inst. Inc., Cary, NC) was used. The initial weight was used in the statistical model as a covariate when significant. *P*-values less than 0.05 were considered significant. The sensory analysis also included the Levene test to verify the variance homogeneity using the "HOVTEST" command.

## Results

### Physicochemical composition

The level of sunflower cake inclusion in the diet did not affect pH (*P* = 0.15), the color parameters lightness (L*; *P* = 0.63), redness (a*; *P* = 0.52), yellowness (b*; *P* = 0.36), saturation index Chroma (C*; *P* = 0.83), or Hue (*P* = 0.20), cooking weight loss (*P* = 0.23) or Warner-Bratzler shear force (*P* = 0.56) of the *longissimus lumborum* muscle of lamb (Table 3).

**Table 3:** Physicochemical composition of the *longissimus lumborum* muscle of Santa Ines lambs fed sunflower cake.

Physicochemical composition	Sunflower cake (% DM)				SEM*	*P*-value^†^	
	0	10	20	30		Linear	Quadratic
pH mean	5.64	5.73	5.80	5.88	0.12	0.15	0.93
Color parameter							
L* (lightness)	38.7	39.2	39.6	37.9	0.99	0.63	0.28
a* (redness)	21.8	22.1	22.7	21.2	0.46	0.52	0.09
b* (yellowness)	6.72	7.24	7.68	7.26	0.46	0.36	0.35
Chroma (saturation)	22.9	23.3	23.5	22.6	0.52	0.83	0.25
Hue (°)	16.9	18.1	19.0	18.6	0.96	0.20	0.46
Cooking weight loss (g/kg)	322	352	334	357	16.3	0.23	0.89
WBSF^¥^ (kgf/cm^2^)	1.49	1.39	1.39	1.42	0.08	0.56	0.47
Moisture (g/kg meat)	757	759	759	772	2.80	<0.01	0.07
Protein (g/kg meat)	194	200	190	201	4.60	0.42	0.40
Ash (g/kg meat)	10.8	10.7	10.9	11.0	0.10	0.27	0.46
Total lipids (g/kg meat)	28.1	32.1	30.7	39.1	1.60	<0.01	0.21
Cholesterol (g/kg fat)	755	663	692	698	14.1	0.61	0.08

*Standard error of the mean.

^†^*P ≤* 0.05.

^¥^Warner-Bratzler shear force.

For the chemical composition of the *longissimus lumborum* muscle, the content of total lipids (*P* <0.01) and moisture (*P* <0.01) increased linearly with sunflower cake inclusion in the experimental diets. Sunflower cake inclusion did not affect the protein (*P* = 0.42), ash (*P* = 0.27) or cholesterol (*P* = 0.61) content.

### Fatty acid profile and nutraceutical parameters

The concentration (g/100 g FAME) of SFA C16:0, total MUFAs (ΣMUFA), and the MUFAs C16:1, C18:1 *trans–11* and C18:1 *cis–9* in the *longissimus lumborum* muscle decreased linearly (*P* <0.01) with the level of sunflower cake in the diet of lambs (Table 4).

**Table 4:** Fatty acid composition (g/100 g FAME) in the *longissimus lumborum* muscle of Santa Ines lambs fed sunflower cake.

Fatty acid (g/100 g FAME)	Sunflower cake (% DM)				SEM^†^	*P*-value^¥^	
	0	10	20	30		Linear	Quadratic
Saturated fatty acids (SFA)							
C10:0 (decanoic acid)	0.14	0.13	0.14	0.13	0.01	0.39	0.79
C12:0 (lauric acid)	0.06	0.10	0.13	0.11	0.01	<0.01	<0.01
C14:0 (myristic acid)	2.29	2.36	2.56	2.22	0.03	1.00	<0.01
C16:0 (palmitic acid)	21.5	20.3	19.1	16.9	0.20	<0.01	0.15
C17:0 (margaric acid)	1.65	1.42	1.46	1.44	0.13	0.34	0.44
C18:0 (stearic acid)	17.4	20.1	22.4	24.2	0.22	<0.01	0.10
Monounsaturated fatty acids (MUFA)							
C16:1 (palmitoleic acid)	4.82	3.5	2.96	2.14	0.10	<0.01	0.03
C18:1 *trans*–11 (vaccenic)	1.12	0.96	0.73	0.75	0.03	<0.01	<0.01
C18:1 *cis*–9 (oleic acid)	39.5	34.7	34.9	32.2	0.43	<0.01	0.02
Polyunsaturated fatty acids (PUFA)							
C18:2 n–6 (linoleic acid)	5.77	9.4	9.07	11.4	0.26	<0.01	0.03
CLA (rumenic acid + isomers)	0.67	1.08	1.08	1.28	0.05	<0.01	0.05
C18:3 n–3 (linolenic acid)	0.67	0.91	0.75	0.86	0.03	0.17	0.06
C20:4 n–6 (arachidonic acid)	3.96	4.48	4.13	5.55	0.09	<0.01	<0.01
C20:5 n–3	0.45	0.56	0.68	0.83	0.04	<0.01	0.68
Group sums and ratios							
ΣSFA	43.0	44.4	45.8	45.0	0.40	<0.01	0.59
ΣMUFA	45.4	39.2	38.6	35.1	0.44	<0.01	<0.01
ΣPUFA	11.5	16.4	15.7	19.9	0.40	<0.01	0.91
ΣPUFA:ΣSFA	0.27	0.38	0.34	0.44	0.01	<0.01	0.80
ΣPUFA:ΣMUFA	0.25	0.42	0.40	0.57	0.01	<0.01	0.62
ΣMUFA:ΣSFA	1.08	0.92	0.86	0.78	0.01	<0.01	<0.01
Σn–6	9.73	13.9	13.2	17.0	0.35	<0.01	0.94
Σn–3	1.12	1.47	1.43	1.68	0.09	<0.01	0.53
n–6:n–3	8.68	9.42	9.25	10.1	1.08	0.72	0.25
Nutraceutical parameters							
Desirable fatty acids	75.5	75.6	77.4	79.2	0.57	<0.01	0.13
Atherogenicity index	0.54	0.52	0.55	0.48	<0.01	<0.01	<0.01
h:H index	1.95	2.10	2.06	2.28	0.01	<0.01	0.02
Δ 9–desaturase C16	18.3	15.8	13.4	11.2	0.40	<0.01	0.78
Δ 9–desaturase C18	70.0	63.3	61.4	57.0	0.17	<0.01	<0.01
Elongase	68.8	71.3	72.5	74.7	0.12	<0.01	0.53

^†^Standard error of the mean.

^¥^*P ≤* 0.05.

The concentrations *(*g/100 g FAME) of SFA C18:0, total PUFAs (ΣPUFA), and the PUFAs C18:2 n–6, CLA, C20:4 n*–*6 and C20:5 n*–*3 increased in the *longissimus lumborum* muscle of lamb meat with sunflower cake inclusion. The sunflower cake inclusion in the lamb diets did not change the fatty acids C10:0 (*P* = 0.39), C17:0 (*P* = 0.34) or C18:3 n*–*3 (*P* = 0.39) in the *longissimus lumborum* muscle. A quadratic effect (*P* = 0.087) was observed for the concentration (g/100 g FAME) of SFA C14:0 in the *longissimus lumborum* muscle from the meat of lambs fed sunflower cake.

The group sums and ratios of ΣPUFA:ΣSFA, ΣPUFA:ΣMUFA, Σn*–*6, and Σn*–*3 and the nutraceutical parameters of desirable fatty acids, hypocholesterolemic and hypercholesterolemic (h:H) fatty acids ratio and elongase activity linearly increased (*P* <0.01) in the *longissimus lumborum* muscle with sunflower cake included in the diet of the lambs. A linear decrease (*P* <0.01) occurred in the ΣMUFA:ΣSFA ratio, Δ*–*9 desaturases C16 and C18 and the atherogenicity index (AI) in the *longissimus lumborum* muscle of lamb. The ratio Σn*–*6:Σn*–*3 in the muscle of lamb was not affected by sunflower cake in the diet.

### Evaluation of sensory attributes

Sunflower cake inclusion in the diet of lambs promoted a linear increase (*P* <0.01) in flavor, tenderness and juiciness of the *longissimus lumborum* muscle (Table 5).

**Table 5:** Sensory attributes of the *longissimus lumborum* muscle of Santa Ines lambs fed sunflower cake.

Attribute*	Sunflower cake (% DM)				SEM^†^	*P*-value^¥^	
	0	10	20	30		Linear	Quadratic
Aroma	7.10	7.10	7.60	7.20	0.20	0.43	0.25
Flavor	6.10	6.80	7.20	7.00	0.23	<0.01	0.05
Tenderness	6.30	7.00	7.00	7.70	0.24	<0.01	0.81
Juiciness	5.40	6.40	6.40	6.70	0.29	<0.01	0.30
Overall acceptance	6.90	6.90	7.00	6.70	0.24	0.59	0.37
Preference^**€**^	12.8	28.2	23.1	35.9	1.54	<0.01	0.44

*Hedonic scale sensory evaluation (1 = dislike extremely and 9 = like extremely).

^†^Standard error of the mean.

^¥^*P*-values were considered significant at 0.05.

^€^Preference values in % of panelists.

For flavor, the scores ranged from "like slightly" to "moderately liked." For tenderness, the scores ranged from “like slightly” to “like very much.” Juiciness scores ranged from “neither like nor dislike” to “like moderately.” The level of 30% sunflower cake inclusion obtained higher scores than lower levels and was more preferred (35.9%) among the panelists. The attributes overall acceptance (*P* = 0.59) and aroma (*P* = 0.43) of the *longissimus lumborum* muscle of lamb meat were not affected by including sunflower cake in the diets of lambs.

## Discussion

The 24 h *post-mortem* pH was similar in the meat from animals in different treatments, which might be related to the similar content of muscle glycogen caused by sunflower cake intake and the reserve mobilization during the development of these animals [33]. The post-mortem curve of pH decline showed that the final pH (24 h after slaughtering) values between 5.64 and 5.88 indicated normal *post mortem* development, leading to a desirable quality of meat [34].

In the present study, sunflower cake inclusion did not affect the color parameters, cooking weight loss or Warner-Bratzler shear force of the lamb meat. Those parameters are among the most important characteristics of meat as the primary attributes considered at the time of purchase [35]. The meat from lambs that ate sunflower cake was considered soft (1.39 to 1.49 kgf/cm^2^) according to Cezar and Sousa [36] who stated that fillets that did not resist a cutting pressure of 2.27 kgf/cm^2^ were considered soft meat. Additionally, the water remaining in meat during cooking directly influences the juiciness, color, and flavor.

The lipid deposition in meat increased with sunflower cake inclusion in the diet of the lambs. These cakes increase the energy density of the ruminant diet and are metabolized and stored in the form of adipose tissue, which affects the meat [9,33]. Despite this increase in lipids, the cholesterol content was not influenced by sunflower cake inclusion. Generally, the proportion of protein and ash remains constant, whereas the proportion of fat increases and that of water decreases [11]. In the present study, fat and moisture contents increased, which were positive factors according to the evaluation of the panelists due to the increase in juiciness (Table 5). The sensation of juiciness is initially determined by the moisture content of the meat, which results in liquid release, and the sensation persists because of the lipid concentration in meat, which stimulates salivation [37,38]. Additionally, the cholesterol concentration ranged from 71.5 to 69.8 mg/100 g of meat, which are low values considered beneficial for the health of consumers [6].

Saturated fatty acid C16:0 content was reduced with the inclusion of sunflower cake in the diet of lambs (Table 2). Additionally, the activity of Δ–9 desaturase C16 in muscle decreased with sunflower cake included in the diets, which was coupled with reduced C16:0 content and that of the precursor C16:1, resulting in decreased concentration of palmitoleic acid [7]. The inclusion of sunflower cake provides a low concentration of propionic acid [39], which is required as an energy source for this biosynthesis. The decrease in concentration of saturated fatty acid C16:0 is beneficial, because this undesirable and hypercholesterolemic FA greatly influences the plasma levels of Low Density Lipoprotein (LDL) and High Density Lipoprotein (HDL) [6,10] and increases the blood cholesterol level by reducing LDL-cholesterol receptor activity and reducing LDL-free space in the bloodstream [40]. Therefore, the reduced concentration of this FA in the *longissimus lumborum* of lambs increases the benefit to human health.

With the addition of sunflower cake in the lamb diet, Stearic acid (C18:0) and nutraceutical parameters, such as CLA, UFA/SFA and PUFA/SFA ratios, n–3 and n–6 concentrations, the hypocholesterolemic:hypercholesterolemic ratio and desirable fatty acids, increased, whereas the atherogenicity index (AI) decreased. The complexity of ruminant digestion is determined primarily by the symbiotic ruminal microbiota, and this complexity will continue to challenge efforts to modulate meat quality, including meat fatty acid composition [7]. Sunflower cake has a relatively high level of linoleic acid (C18:2 n–6) as a self-defense mechanism. The ruminal bacteria *Butyrivibrio fibrisolvens* and *Butyrivibrio proteoclasticus* conduct the biohydrogenation process and break the unsaturation to produce less toxic FAs [37], resulting in stearic FA production [41] and CLA, which is an intermediate product of incomplete biohydrogenation of unsaturated fatty acids (C18:1 *trans*–11 and C18:1 *cis*–9). CLA can also be produced endogenously through the desaturation of trans-vaccenic fatty acid (C18:1 *trans*–11) by the enzyme stearoyl-CoA desaturase or Δ–9 desaturase C18 [37]. In the present study, the concentration of CLA increased when the content of C18:1 *trans*–11 decreased. The products of biohydrogenation are beneficial for human health; C18:0 is a SFA but has a neutral effect because of the endogenous conversion to oleic acid (C18:1 *cis*–9) and reduction of serum cholesterol [42].

The concentrations of arachidonic (C20:4 n–6) and eicosapentaenoic (C20:5 n–3) fatty acids increased with the inclusion of sunflower cake in lamb diet. These fatty acids are synthesized on the surface of the smooth endoplasmic reticulum from the precursors linoleic acid and linolenic acid, respectively, through elongation (elongase enzyme) and desaturation (desaturase enzymes) processes of the carbon chain [7]. The elongase enzyme functions with fatty acid desaturases to generate many of the long-chain mono- and polyunsaturated fatty acids such as palmitic, palmitoleic and oleic acids that are assimilated into cellular lipids. [6]. In animals fed higher levels of sunflower cake, the low amounts of oleic acid in the muscle of animals reduced activity of the Δ9 desaturase enzyme C18. However, because unsaturation increases with sunflower cake in the diet, oxidative stability is likely impaired and off flavors could develop in the meat during storage, in addition to a likely increase in a warmed-over flavor [43–44].

The AI assesses plaque formation capacity in the blood vessels. At low values, more anti-atherogenic FAs are in the lipids, which can contribute to prevention of cardiovascular diseases [28]. In this research, the average AI ranged from 0.55 to 0.48, which is less than the average found by Ulbricht and Southgate [27] for lamb meat (1.00).

Sunflower cake inclusion did not affect (*P* > 0.05) the overall acceptance or aroma of the meat. These sensorial characteristics were scored as "like moderately." Based on the scores of the panelists, all the meats were well accepted. However, sunflower cake inclusion in the diet of lambs gave better flavor, tenderness, and juiciness to the meat. Thus, the meat of the animals that received 30% sunflower cake inclusion was preferred (35.9%). The meat aroma and flavor are directly related to the FA profile of the meat [7], particularly the PUFA content, which provide better flavor. Although sunflower cake inclusion did not affect the aroma of lamb meat, according to the panelists, meat quality improved measured by flavor, most likely because the inclusion of sunflower cake increased the concentration of polyunsaturated fatty acids in the meat [7,38].

## Conclusions

Sunflower cake up to 30% of total DM can be included in the diet of lambs to increase lipid nutritional quality and the sensory experience of lamb meat, in addition to improving levels of nutraceutical parameters, such as content of CLA, the AI, PUFA:SFA and PUFA:MUFA ratios, desirable fatty acids and the h:H ratio, which are beneficial to consumer health.
